# Reconstruction of a Severe Open Tibiofibular Fracture using an Ipsilateral Vascularized Fractured Fibula with a Thoracodorsal Artery Perforator Free Flap

**DOI:** 10.1055/a-2119-3575

**Published:** 2023-10-05

**Authors:** Lan Sook Chang, Dae Kwan Kim, Ji Ah Park, Kyu Tae Hwang, Youn Hwan Kim

**Affiliations:** 1 Department of Plastic and Reconstructive Surgery, College of Medicine, Hanyang University, Seoul, Korea; 2 Design Laboratory of the Technology Commercialization Center, Industry-University Cooperation Foundation of Hanyang University, Seoul, Korea; 3 Department of Orthopaedic Surgery, College of Medicine, Hanyang University, Seoul, Korea

**Keywords:** perforator flap, free tissue flaps, fibula, tibia

## Abstract

The Gustilo IIIB tibiofibular fractures often result in long bone loss and extensive soft tissue defects. Reconstruction of these complex wounds is very challenging, especially when it includes long bone grafts, because the donor site is limited. We describe our experience using a set of chimeric ipsilateral vascularized fibula grafts with a thoracodorsal artery perforator free flap to reconstruct the traumatic tibia defects. A 66-year-old male suffered a severe comminuted tibia fracture and segmented fibula fracture with large soft tissue defects as a result of a traffic accident. He also had an open calcaneal fracture with soft tissue defects on the ipsilateral side. All the main vessels of the lower extremity were intact, and the cortical bone defect of the tibia was almost as large as the fractured fibula segment. We used an ipsilateral vascularized fibula graft to reconstruct the tibia and a thoracodorsal artery perforator flap to resurface the soft tissue, using the distal ends of peroneal vessels as named into sequential chimeric flaps. After 3 weeks, the calcaneal defect was reconstructed with second thoracodorsal artery perforator free flap. Reconstruction was successful and allowed rapid rehabilitation because of reduced donor site morbidity.

## Introduction

Treatments of traumatic long bone loss in the lower extremity can be categorized into three groups. 1 2 3 4 5 The first is one-stage reconstruction using free vascularized fibular grafts and the second a two-stage reconstruction referred to as the induced membrane technique, while the third consists of bone transfer using an external fixator. The advantages and disadvantages of these treatments have been well described in numerous reports. 1 2 3 4 5 Nonetheless, when treating traumatic long bone loss, all factors including bone length, patient compliance, age, and soft tissue condition, etc. should be considered in each case.

A Gustilo IIIB open tibiofibular fracture often results in tibia bone loss due to severe comminuted and contaminated fractured segments that have to be removed to prevent infection. 6 7 8 Fibula fracture is often not as severe as tibia fracture, because the fibula is a deep structure surrounded by muscle, whereas a lot of energy is absorbed by the tibia due to its more anterior and superficial location. The peroneal vessels are often saved for the same reason.

The Gustilo IIIB open fracture is also accompanied by extensive soft tissue defects, in addition to the underlying bony defects. Reconstruction of this complex extremity defect often exploits the concept of the chimeric flap, which enables the use of flaps combining different tissue components to be used. 9 Among the categories of chimeric flaps, prefabricated chimeric flap is created by adding other components to a simple flap by microanastomosis to a proximal branch or to the distal end of an axial vessel. 10 11 This type of flap has previously been described by different names, such as sequential flap or bridge flap. 12

We had a very interesting case of a Gustilo–Anderson IIIB open tibiofibular fracture. The patient suffered a severe comminuted tibia fracture and a segmented fibula fracture with large soft tissue defects. Fortunately, all the vessels of the lower extremity including the peroneal vessels were intact and the tibial cortical bone defect was almost the same size as the fibula fracture segment. Hence, we used an ipsilateral vascularized fibula graft for tibia reconstruction. We also employed a thoracodorsal artery perforator free flap for resurfacing the soft tissue using the distal end of the peroneal vessels for the sequential chimeric flap.

Here, we present our clinical experience using a sequential chimeric type of flap to reconstruct an open tibiofibular fracture.

## Case

A 66-year-old male without underlying disease suffered a pedestrian traffic accident that resulted in a comminuted segmental tibia fracture and segmental fibula fracture with soft tissue defects as Gustilo–Anderson type IIIB fracture on his left lower leg. Unfortunately, he also suffered open calcaneal fracture with soft tissue defects on the ipsilateral side. An emergent operation was performed in a local clinic. Wiring of the bone fragment and application of an Ilizarov external fixator were performed, but the patient was referred to our trauma unit 7 days after the injury to cover the extensive soft tissue defect.

**Fig. 1:**
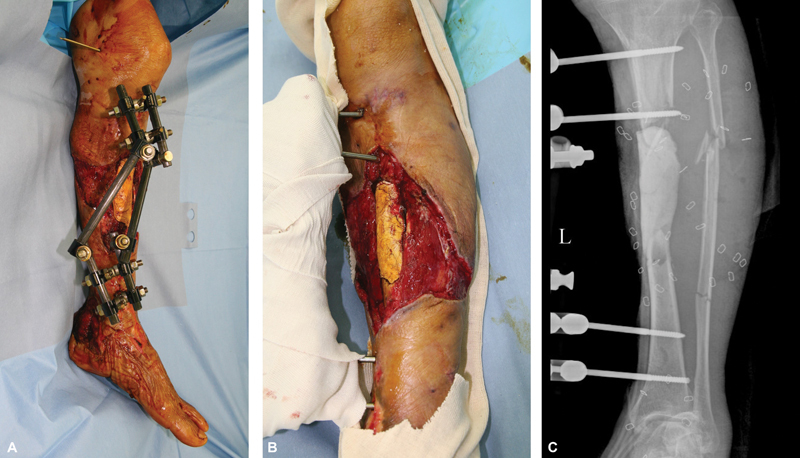
Necrotic tissues were debrided including proximal tibia bone and soft tissues. External fixator system was applied ( **A** ). Bone cement was inserted where the tibia defect was ( **B** ). Cortical bone defects of tibia and segmental fracture of fibula was revealed in simple X-ray after three times debridement ( **C** ).

The orthopaedic surgery team removed all the dead bone including the fragmented tibia, which was fixed with wire, and debrided the necrotic soft tissues. The 11-cm tibia defect was filled with bone cement and an external fixator was applied for stabilization ( Fig. 1 ). Serial irrigation and debridement were performed three times at intervals of 1 week in the operating room. Wound cultures were all negative, and there was no clinical evidence of infection.

**Fig. 2:**
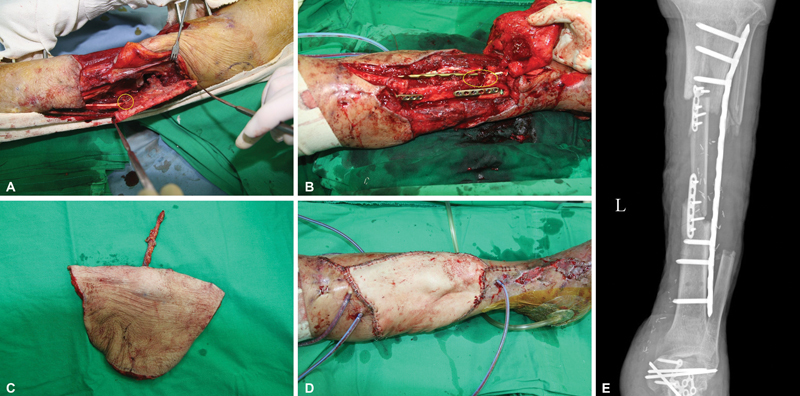
Ipsilateral vascularized fibula graft approximately 12.5 cm size was harvested. The yellow circle indicates the distal stump of the peroneal recipient vessel. Distal peroneal vessels were ligated with hemoclip ( **A** ). Orthopaedic surgery team removed bone cement spacer and converted external fixator to internal fixation with plates and screws at lateral side of proximal tibia. Transferred fibula was rotated approximately 90 degrees in clockwise and fixed to medial side of tibia using 6-hole plates and screws at both docking sites. The yellow circle indicates roughly the location of the anastomosis site under the lateral plate of the tibia ( **B** ). A 22 cm × 14 cm sized thoracodorsal artery perforator flap was harvested ( **C** ). Immediate postoperative view showed good contour on proximal tibia area ( **D** ). Plain X-ray revealed that the transferred fibula was inserted well in the defect area of the tibia ( **E** ).

**Fig. 3:**
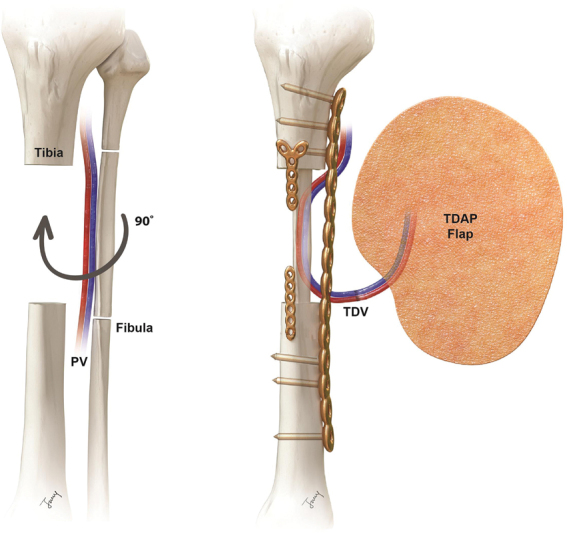
Illustration of sequential chimeric type of ipsilateral vascularized fractured fibula graft with thoracodorsal artery perforator free flap. The fibula fragment was rotated approximately 90 degrees clockwise around its long axis.

After wound stabilization and confirmation that there were no signs of infection, reconstruction of the bone and soft tissue was planned, with close discussion between the orthopaedic surgery and plastic surgery teams. In the preoperative computed tomography angiography, fortunately, all the main vessels of the lower extremity were found to be intact. First, the plastic surgery team harvested an ipsilateral vascularized fibula graft of approximately 12.5 cm size which could be harvested in the fractured area ( Fig. 2 ). Fortunately, the fractured segment of the fibula could be easily handled without cutting the distal or proximal fibula because the distal fibula was already dislocated, and we just had to cut the proximal fibula around the fracture area. We were also able to save the distal peroneal vessels that were 2 cm longer than the fibula bone graft when we ligated the distal peroneal vessels. These vessels were used as recipients for the flap, to cover the soft tissue defect. The harvested fibula was transferred to the damaged area of the proximal tibia beneath the tibialis anterior muscle. The orthopaedic surgery team removed the bone cement spacer and converted the external fixation to internal fixation using plates and screws on the lateral side of the proximal tibia. The transferred fibula graft was fixed to the medial side of the tibia with 6-hole plates and screws at both docking sites, and the peroneal vessels of the transferred fibula were positioned beneath the lateral reconstructed plate. In addition, a small piece of tibialis anterior muscle was removed to obtain microanastomosis fields and prevent compression of the pedicle. The plastic surgery team harvested a 22 × 14 cm thoracodorsal artery perforator flap from the right lateral thoracic region with the patient in a supine position. This flap was used to cover the soft tissue defect in the anterior tibial area. The thoracodorsal vessels were anastomosed with preservation of the distal peroneal vessels ( Fig. 3 ). The flap survived without complications and all the wounds stabilized except the calcaneal wound.

Calcaneal reconstruction was performed 3 weeks after the proximal tibia reconstruction, and during this time the calcaneal wound was maintained using a negative pressure wound therapy (NPWT) system. The orthopaedic surgery team performed open reduction and internal fixation with plates and screws, and a Steinman pin, after which the plastic surgery team resurfaced the calcaneal defects area using a 17 cm × 15 cm sized thoracodorsal artery perforator flap from the left lateral thoracic region. The thoracodorsal vessels were anastomosed to posterior tibial vessels. This second flap also survived, and the patient recovered well without any acute complications.

Loosening of the proximal fibula fixation due to a docking site fracture was found at postoperative 2 months. We elevated the previous flap on the medial side while preserving the membrane and the orthopaedic surgery team exchanged the original proximal plate and screws with larger and longer ones. Additionally, nonvascularized iliac cancellous bone grafts were performed at both docking sites to enhance bone union according to the induced membrane technique.

**Fig. 4:**
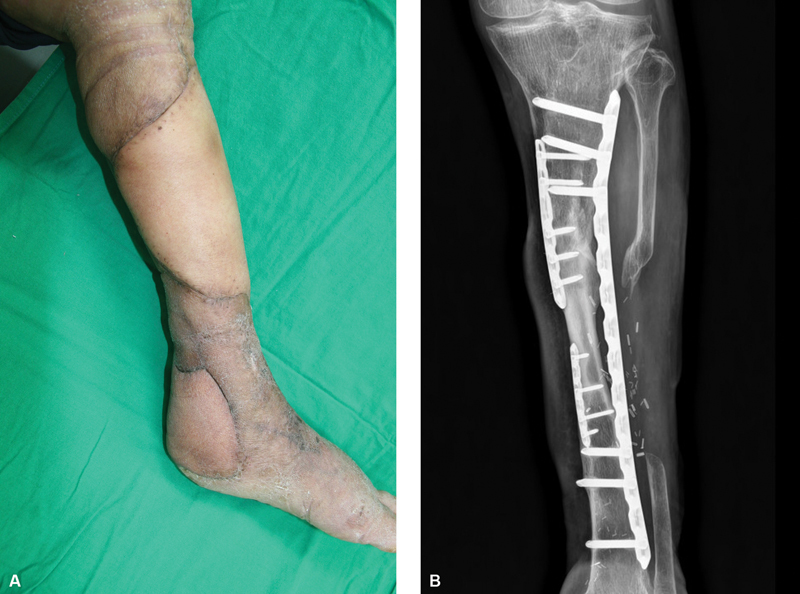
Long-term follow-up view showed good contour without any debulking procedures ( **A** ). Bone union proceeded well and the transferred fibula had hypertrophied by 3 months later ( **B** ).

Bone union was successful, and a postoperative 6-month X-ray revealed hypertrophy of the transferred fibula. The whole soft tissue defect was covered without any wound problem and the patient could walk unaided ( Fig. 4 ).

## Discussion

Our patient suffered Gustilo–Anderson type IIIB open fractures at two sites, including the proximal tibia and calcaneal region. There was a high probability that this severe injury would require amputation, so we hesitated to use the opposite leg as a donor because of concern that the patient might end up with difficulty to use both legs. In addition, reconstruction of the injury required both a bony component and extensive soft tissue coverage. The contralateral vascularized fibula osteocutaneous flap was unsuitable because of its limited flap dimensions.

Fortunately, the length of the fibula fracture fragment (12.5 cm) matched the length of the tibia cortical defect (11 cm), and the peroneal vessels were uninjured due to being surrounded by deep structures of the lower extremity. An ipsilateral vascularized fibula flap can be used to reconstruct tibial defects, without donor site morbidity. In addition, it can be used as a sequential chimeric flap concept, to deal with complex defects by microanastomosis of a large thoracodorsal artery perforator flap to the distal end of the peroneal vessel. 9 13 14 In fact, peroneal vessels are not favored as primary choice of recipient because of their deep structure. The distal stump was short and we also had difficulty positioning the vessel for microanastomosis after bone fixation. To solve this problem, we rotated the fibula 90 degrees and located the peroneal vessels between the lateral tibia plate and the locking plate of the fibula fixation plate. The other main artery, which was conserved in this process, could be used later as a recipient for calcaneal resurfacing, thus enabling economical use of the limited number of vessels in the injured lower extremity.

Since Masquelet and colleagues described it in 1986, the induced membrane technique has become popular for treating traumatic long bone defects. 2 3 4 5 We have often used it to treat chronic osteomyelitis or long bone defects. Its simplicity is a powerful advantage and the higher union rate with less donor morbidity makes it attractive compared with other techniques. In this case, a one-stage vascularized fibula graft was attempted using the induced membrane technique, but unfortunately, docking site nonunion was found. While saving the membrane, we changed the plates and screws and inserted a cancellous iliac bone graft at the proximal and distal docking sites between the transferred fibula and tibia. Reelevating thoracodorsal artery perforator flap while saving the membrane was easy because of the skin flap characteristics. Skin flaps or fasciocutaneous flaps are much more suitable for secondary procedures than muscle flaps for treating Gustilo–Anderson type IIIB open tibia fractures. 15 16 Bone union proceeded well and the transferred fibula had hypertrophied by 3 months later.

Early rehabilitation was achieved without using the contralateral leg, so the patient could walk comfortably with a crutch. Moreover, donor site morbidity was insignificant due to the use of two perforator flaps from the lateral thoracic region without sacrificing any muscle.

Several lessons were learned from this case. The first was that all procedures should only be performed after full discussion between the reconstructive surgeons. This means that a multidisciplinary team approach is very important. We were able to save the limbs thanks to new procedures such as the use of the fractured segment of fibula, which was an idea derived from close discussion between the orthopaedic and plastic surgeons. The second lesson was that one procedure is not enough for a severe Gustilo–Anderson type IIIB open fracture. Combination techniques should be considered, such as sequential chimeric free flaps, one-stage vascular fibular grafts, the induced membrane technique, and the fix and flap technique with bridging therapy using NPWT. The third lesson was that we should always think about future rehabilitation of multiple open fractures. Donor site morbidity should be considered before the final reconstruction. A fourth lesson is that we should consider that there is always a way to save the leg. In some situations, the use of an ipsilateral fractured fibula transfer along with a sequential chimeric flap may offer another option for specific severe trauma cases.

Grafting from the ipsilateral fractured fibula with sequential chimeric flaps may allow more rapid rehabilitation of patients because reconstruction is faster and donor site morbidity is reduced. This procedure may provide a good option for Gustilo IIIB tibiofibular fractures in special circumstances.
